# THE ASSOCIATIONS OF ENERGY CONSUMPTION AND EXPENDITURE WITH SELF-PERCEIVED WELL-BEING AMONG CHINESE ADULTS

**DOI:** 10.1093/geroni/igad104.2680

**Published:** 2023-12-21

## Abstract

**Background and Objectives:**

Households in China have gradually moved to use of cleaner fuels, but the proportion using solid fuels in daily life remains as high as 30%, resulting in approximately 420,000 premature deaths annually. Energy use and its impact on health may differ by age, as less active lifestyle in later life may lead to increased household energy consumption aggravating poor health consequences. The twofold aims of this study are: 1) to examine energy consumption patterns and expenditure between young Chinese (aged 18-59) and older Chinese (aged 60 or above), and 2) to identify how energy consumption patterns and expenditure are associated with self-perceived wellbeing in the two respective age groups.

**Methods:**

A total of 2,303 valid samples (M age = 51, SD = 16.2) were obtained from Chinese General Social Survey (CGSS) in 2015. Hierarchical linear modelling analyses were employed using HLMs 6.08 for each age group to examine the influence of individual-level factors and cluster-level covariates (i.e., environmental pollution, health care resources, and economic development at provincial level).

**Results:**

Among the young adults, those using oils, gas, and electricity reported better self-perceived wellbeing than those using solid fuels. Among the older adults, reliance on fuel gas and energy expenditure were associated with better self-perceived wellbeing.

**Conclusions:**

The harnessing of cleaner energy has the potential to increase subjective wellbeing across age groups. For older adults, the higher energy expenditure might indicate a dormant or isolated lifestyle, which calls for practice interventions to improve social engagement and promote wellbeing.

